# A global bibliometric analysis on Kawasaki disease research over the last 5 years (2017–2021)

**DOI:** 10.3389/fpubh.2022.1075659

**Published:** 2023-01-10

**Authors:** Weifu Tan, Liao Jing, Yunxiao Wang, Wei Li

**Affiliations:** ^1^Department of Neonatology, The First Clinical Medical College of Jinan University, The First Affiliated Hospital of Jinan University, Guangzhou, China; ^2^Department of Neonatology, The Dongguan Affiliated Hospital of Medical College of Jinan University, The Binhaiwan Central Hospital of Dongguan, Dongguan, China

**Keywords:** Kawasaki disease, COVID-19, Web of Science, Scopus, bibliometric analysis

## Abstract

**Background:**

Kawasaki disease (KD) is a systemic vasculitis of unknown etiology that mainly affects children. We aim to conduct a bibliometric analysis to explore the latest research hotspots and trends of KD.

**Method:**

By using the keywords “Kawasaki disease,” “Kawasaki syndrome”, and “Mucocutaneous Lymph Node Syndrome,” the Web of Science (WOS) and Scopus databases were searched for publications related to KD from 2017 to 2021. Author, country and journal submissions were classified and evaluated using Biblioshiny software (using R language). VOSviewer (version 1.6.18) was used to visualize the relevant network relationships.

**Result:**

According to the search strategy, 5,848 and 6,804 KD studies were published in WOS and Scopus, respectively. The results showed an overall increasing trend in the number of publications and citations during the study period. The three most influential institutions in the WOS were St. Marianna University, Kawasaki Medical School and The University of Tokyo in Japan, while in Scopus, Harvard Medical School, University of California and Tehran University of Medical Sciences were the most influential institutions. The most influential authors of the two databases are Goodman SG, Kazunori Kataoka, and Takeshi Kimura of the WOS and Marx Nikolau and Wang Y, Burns JC, and Newburger JW of the Scopus, respectively. And Scientific Reports and Frontiers in Pediatrics were the most critical journals. The most cited documents were the WOS document by McCrindle et al. and the Scopus document by Benjamin et al. published in 2017, while the keywords in the last few years were focused on “COVID-19,” “multisystem inflammatory syndrome,” and “pandemic.”

**Conclusion:**

This bibliometric analysis summarizes for the first time the research progress in KD (2017–2021), providing a qualitative and quantitative assessment of KD research bibliometric information. In the field, researchers mainly from Japan and USA are dominant, followed by China. It is recommended to pay close attention to the latest hot spots, such as “COVID-19” and “multisystem inflammatory syndrome.” These results provide a more intuitive and convenient way for researchers to obtain the latest information on KD.

## Background

Kawasaki Disease (KD) was first discovered in Japan in 1967 and subsequently reported in dozens of countries around the world. KD is mainly characterized by systemic inflammation, affecting children under 5 years of age, and the incidence of KD has racial and seasonal differences (1). KD can be triggered by infection of various pathogenic microorganisms (2). Genetics may also play an important role in the pathogenesis of KD (3). Studies have found that genetic abnormalities in several different functional types of genes may increase the risk of KD, for example, enhancing T cell activation (ITPKC, ORAI1, and STIM1), dysregulation of B cell signaling (CD40, BLK, and FCGR2A), inhibiting apoptosis (CASP3), and changing transforming growth factor-β signaling (TGFB2, TGFBR2, MMP, and SMAD), etc. (4). The clinical diagnosis of classical KD requires the presence of at least four of the following five clinical features when fever of at least 5 days is satisfied: (i) diffuse congestion and chancre in the lips and mouth; (ii) bilateral conjunctiva in a congested state without exudates; (iii) rash including maculopapular rash, erythema multiforme, etc.; (iv) erythema of the hands and feet in the acute phase and/or subacute peri-finger and toe molting; and (v) enlarged lymph nodes (at least 1.5 cm in diameter), mostly unilateral (5).

With the epidemic of COVID-19, a Kawasaki-like disease has gradually attracted the attention of clinicians and scientists (6, 7). Guidance from the Royal College of Pediatrics and Child Health states that children with (i) persistent fever >48 h, lymphomegaly and evidence of single or multi-organ dysfunction; and (ii) exclusion of any other microorganism causing the disease can be considered for the diagnosis of KD-like multi-inflammatory syndrome (8). Some of the SARS-CoV-2 infected children diagnosed with multi-system inflammatory syndrome (MIS) exhibit clinical features that meet the criteria for Kawasaki disease (9). The first major UK case series study on children with MIS found that about 10–20% of children were eligible for Kawasaki disease diagnosis (10). In contrast, the results of an Italian national multicenter survey showed that children with Kawasaki-like disease had a higher rate of positive SARS-CoV-2 detection (75,5%) compared to children with KD (11).

### Research gaps based on literature review

Integrating and analyzing the scientific results of a field of study has always been a priority for scientists, and this has given rise to systematic reviews and meta-analyses, a method of data analysis that integrates different results of similar studies. Using the year of publication as a cut-off point provides a more convenient and macroscopic view of the focus of Kawasaki disease research in each year. A search of the PubMed database revealed several systematic reviews and meta-analyses of research on KD published between 2017 and 2021.

A systematic review assessing the application of steroids for coronary complications in KD was published online by the Cochrane Collaboration in 2017 (12). The study included a total of seven high-quality randomized controlled studies and noted that steroid application in the acute phase of KD improved coronary lesions and significantly reduced inflammatory marker levels. And people in Asia, with high-risk scores and prolonged steroid application, are more likely to be benefiting from steroid use. The study was resubmitted in an updated version in 2022. This updated systematic review included 1 new randomized controlled study in addition to the original study and concluded that the use of corticosteroids also reduced the duration of clinical symptoms (13).

Li et al. (14) published a meta-analysis of predictors of intravenous immunoglobulin-resistant (IVIG-resistant) KD in children in 2018, which included 28 studies totaling 4,442 children with IVIG-resistant KD. This meta-analysis summarized the clinical characteristics of all children as well as certain laboratory test indices. Ultimately, the initial administration of IVIG within 4.0 days after the onset of symptoms, increased clots in blood sedimentation, decreased hemoglobin and platelet counts, oral mucosal lesions, enlarged cervical lymph nodes, swollen extremities and polymorphic rash were found to be risk factors for IVIG resistance. In contrast to previous meta-analyses predicting KD risk factors, this study newly added clinical characteristics, duration of initial drug administration, and hemoglobin concentration as risk factors involved in resistant KD. In the same year, Xie et al. (15) identified new potential genetic biomarkers of KD by meta-analysis, in which 62 genes including genetic polymorphisms of ACE, BLK, CASP3, CD40, FCGR2A, FGβ, HLA-E, IL1A, IL6, ITPKC, LTA, MPO, PD1, SMAD3, CCL17, and TNF might be associated with KD susceptibility, and genetic variants in 47 genes including BTNL2, CASP3, FCGR2A, FGF23, FGβ, GRIN3A, HLA-E, IL10, ITPKC, and TGFBR2 may be associated with the incidence of coronary artery lesions in KD.

In 2019, Tanoshima et al. (16) included 20 clinical studies and attempted a systematic review and meta-analysis in order to assess the effectiveness of antiplatelet agents in the treatment of KD. The antiplatelet agents included in the studies were mainly aspirin, flurbiprofen, dipyridamole, and choline salicylate. However, quantitative synthetic analysis was ultimately not performed due to the high heterogeneity of the included studies and the lack of quantitative data. This study concluded that although the application of antiplatelet agents can inhibit platelet aggregation, strong evidence for the effectiveness of antiplatelet therapy is lacking. In addition, to clarify the efficacy and safety of TNF-α blockers (i.e., infliximab and etanercept) for the treatment of children with KD, a new systematic review article was published online by the Cochrane Library in 2019 (17). The study conclusively determined that lower quality evidence suggests that the application of TNF-α blockers is beneficial in drug-resistant KD. However, due to the lack of larger, high-quality studies, the above conclusions need to be treated with caution. Meanwhile, another systematic review was conducted by Crayne et al. (18) on the efficacy and safety of second-line therapy (including a second intravenous IVIG, methylprednisolone and infliximab) in patients with refractory Kawasaki disease in patients with drug-resistant KD. This study concluded that infliximab monotherapy should be considered more as second-line therapy in children who do not respond to first IVIG treatment and that it is more effective than second IVIG in relieving febrile symptoms in children.

Two systematic reviews exploring the dose of aspirin therapy in KD were published online in 2020, respectively (19, 20). Interestingly both studies suggest that the application of low doses of aspirin in the acute phase of KD may benefit the child in the treatment of KD. Jia et al. (19) concluded that the application of low-dose aspirin is comparable to high-dose aspirin therapy with less severe side effects, while Chiang et al. (20) stated that prescribing low or no aspirin in the acute phase of KD was strongly associated with a low incidence of coronary lesions. To clarify the influence of genetic factors on KD susceptibility, Ferdosian et al. (21, 22) conducted a meta-analysis from IL-10 polymorphisms, TNF-α rs1800629, CASP3 rs72689236 and FCGR2A rs1801274 polymorphisms, respectively. The results showed that the IL-10-592 A > C polymorphism, CASPS rs72689236 and FCGR2A rs1801274 polymorphisms may mediate the susceptibility of individuals to KD. Another meta-analysis has also indicated that the CD32a polymorphism rs1801274 is strongly associated with KD pathogenesis and its A allele influences the incidence of KD (23).

The SARS-CoV-2 outbreak epidemic in 2019, with much clear evidence of its association with KD, has generated interest among researchers to link and further analyze both SARS-CoV-2 infection and KD. To clarify the clinical features and investigate the pathogenesis of pediatric multisystem inflammation syndrome (PMIS), Zou et al. (24) searched PubMed and Embase for relevant cases and performed a meta-analysis. This study found that PMIS usually had prolonged fever, gastrointestinal symptoms, cardiogenic shock and Kawasaki-like syndrome, and that SARS-CoV-2 infection was associated with a significant inflammatory state. Abrams et al. (25) published a systematic review with similar findings, which suggested that the vast majority of the 440 children with MIS included presented with gastrointestinal, skin/mucosal symptoms and cardiovascular symptoms, with significantly elevated levels of laboratory tests for inflammatory markers such as C-reactive protein, interleukin-6 and fibrinogen. Lamrani et al. (26) published a case series meta-analysis titled Kawasaki Disease Shock Syndrome vs. Classical Kawasaki Disease: a Meta-analysis and Comparison With SARS-CoV-2 Multisystem Inflammatory Syndrome in 2021 and first explored the differences between KD shock syndrome and traditional KD. This study found that children with KD shock syndrome were characterized by older age, higher inflammatory index C-reactive protein, higher odds of intravenous immunoglobulin resistance, longer hospital stay, and higher rate of coronary artery abnormalities compared to children with classical KD. In addition, it has also been suggested that SARS-Cov2 can act as a trigger that can lead to a second recurrence of KD in genetically susceptible individuals (27).

It can be found that the current KD research mainly focuses on the etiological mechanisms of the disease, therapeutic measures, and the relationship with SARS-CoV-2 infection. Although the above systematic reviews and meta-analyses synthesize some of the research findings in the field of KD research from different perspectives, they do not provide a more comprehensive picture of the current status of research in the field of KD and the changes in hot spots. Therefore, it has become an urgent scientific problem to conduct research on the current status and hot trends of KD as an object of inquiry.

### Research objectives and expected contributions

Bibliometric analysis is a tool for analyzing published academic publications and the development trends and hot spots of a research field through data statistics (28). By analyzing indicators such as the number of citations, the author's work list, national or thematic bibliography, and publication mode, the leading direction of the research field and the institutions and scholars who have found the most research output can be determined (28, 29). There is no denying that bibliometric analysis is widely used in many different disciplines, including economics and medicine. KD has always been a disease that clinicians and scholars pay close attention to.

However, there has been no bibliometric analysis and summary of the research publications on KD in the past 5 years. Therefore, this study aims to provide a comprehensive understanding of the global trends in KD research over the past 5 years by using a bibliometric approach, using the Clarivate Analytics Web of Science (WOS) database and Elsevier's Scopus database as literature sources, to evaluate the current state of research and expect to identify current research hotspots and frontiers.

## Method

### Data collection

Electronic literature search was performed through the Wed of Science (WOS) and the Scopus database, respectively. The search strategies for the different databases are shown below: WOS: Topic = (“Kawasaki disease”) OR Topic = (“Kawasaki syndrome”) OR Topic = (“Mucocutaneous Lymph Node Syndrome”); Scopus: ALL (“Kawasaki disease”) OR ALL (“Kawasaki syndrome”) OR ALL (“Mucocutaneous Lymph Node Syndrome”). The time range is set from January 1, 2017 to December 31, 2021. The literature search and data collection time were August 15, 2022. The document language is limited to English. All bibliometric data ultimately included in the analysis were recorded in plain text format and cited references were exported from WOS/Scopus. The export file contains all the extracted information about the type and main content of the study. The literature search was carried out by two authors independently, and then the results of the two were compared. If there were any differences, the authors discussed with the third independent author and selected the optimal results.

### Data cleaning

The collected literature data were first imported into Citespace (version 6.1.R2), and the duplicate literature was removed, as well as the conference abstracts and non-research articles (including editorials, abstracts, letters, news and newsletters, etc.). Finally, 5,848 and 6,804 documents were obtained from WOS and Scopus, respectively.

### Data analysis

Bibliometrix Biblioshiny R-Package software (https://bibliometrix.org/biblioshiny/biblioshiny1.html) is used to analyze bibliometric data and VOSviewer is used (version 1.6.18) visualize the relevant network relationships (30, 31). In addition, Microsoft Excel 2019 was used to draw graphs and make data tables. Bibliometric data, including title of research paper, number of citations, publication year, author identity, author's country, publishing institution and keywords, etc.

This study analyzed the number of papers published and the publication trend of this research topic in the past 5 years. The top journals with the highest number of published papers, the most cited journals and the top 5 most cited articles were analyzed. The most influential research institutions and countries were analyzed based on the highest number of papers published on the research topic during 2017–2021. The top 10 most influential and productive authors were also analyzed and the co-citation author network and author collaboration network were mapped, clustered as “Leading Eigenvalues,” based on a higher “Betweenness.” The *h*-index was selected to evaluate the scientific influence of research authors/literature journals (32). And h-index is generated through Bibliometrix Biblioshiny analysis of bibliometrics data. International cooperation in the top 10 countries for Kawasaki disease research was quantified using Multi-country Publications (MCP) and Single Country Publications (SCP) scales. In addition, VOSviewer generates a national collaboration network to visualize research collaborations between countries. The keywords of the literature on this research topic were analyzed and word cloud maps were drawn to visualize the weight of keywords plus. Also, a graph of three domains was constructed in order to observe the inflow and outflow between the top 12 authors, the top 15 authors keywords and the top 10 countries that contributed to Kawasaki disease research in the last 5 years.

## Result

### Search result

Initially, 7,032 and 8,661 documents were obtained from WOS and Scopus, respectively. According to the inclusion and exclusion criteria, after excluding 1,183 ineligible articles and 1 duplicate article from the WOS database search, 5,848 studies were retained, including 5,102 Articles (87.24%) and 746 Reviews (12.76%); after excluding 1,857 ineligible or duplicate documents, the Scopus database finally 6,804 studies were retained, which included 5,154 Articles (75.75%) and 1,650 Reviews (24.25%). All bibliometric information of the above screened literature was further analyzed.

### Major characteristics of the included studies

#### WOS database

The 5,848 included documents originated from 1,793 different journals and were published by 56,890 authors (the average citation per document and per document per year were 2.62 and 13.82), of which 103 were single-author documents. The average growth rate of published articles per year is 19.83%. Japan was the country with the highest total citations (40.75%), followed by the United States (23.81%) and the United Kingdom (6.35%). On the other hand, the country with the highest average number of citations for papers was Switzerland (51.09).

#### Scopus database

The 6,804 included studies were derived from 2,124 journals and published by 32,762 authors (mean 7.43 authors per paper). Of these, 295 were single-author literature, authored independently by 245 authors. The average annual growth rate of published articles is 24.05%. In the Scopus database, the United States is the country with the highest total citations (21.63%), followed by China (10.17%) and Germany (5.98%), while Japan is in 9th place in the world with a citation rate of 3.96%. In addition, the country with the highest average citations was Germany with 44.89.

### Global trend of publication and citation

**Figure 1:**
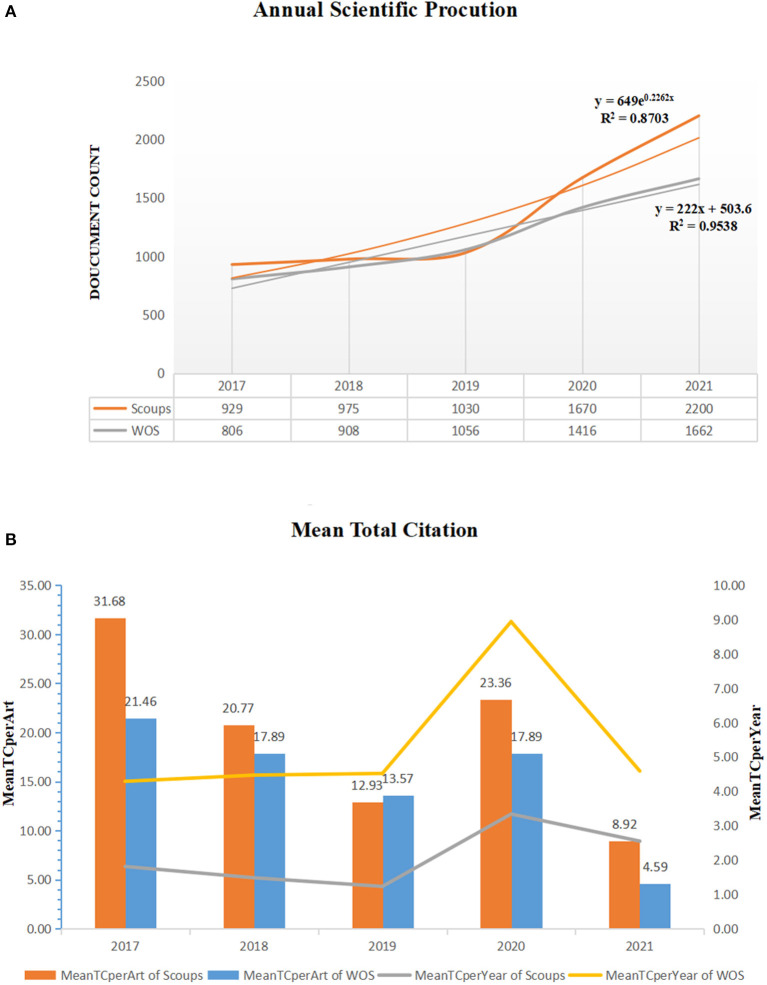
Global publication trends **(A)** and citation trends **(B)** for KD research. The left vertical coordinate indicates the average number of citations per document, and the right vertical coordinate indicates the average number of citations per year.

In the last 5 years (2017–2021), t the number of publications and citations of KD research are continuing to grow, with slightly inconsistent growth trends for the two databases. The publication and citation trends for the WOS and Scopus databases are shown in Figure 1. Fitting curves between time and the number of published studies per year, where Scopus and WOS used exponential and linear functions, respectively. And their *R*-squared values were 87.03 and 95.38%, respectively. The average citations and average annual citations of studies published in the two databases from 2017 to 2021 are shown in Figure 1B. The average citations of the studies published in Scopus were higher than the average citations of WOS's; for the citation trend curve, both databases showed a significant increase in citations of documents in 2019–2020, followed by a drop in both.

### Most productive journals, most cited journals and journal publishing trends

**Figure 2:**
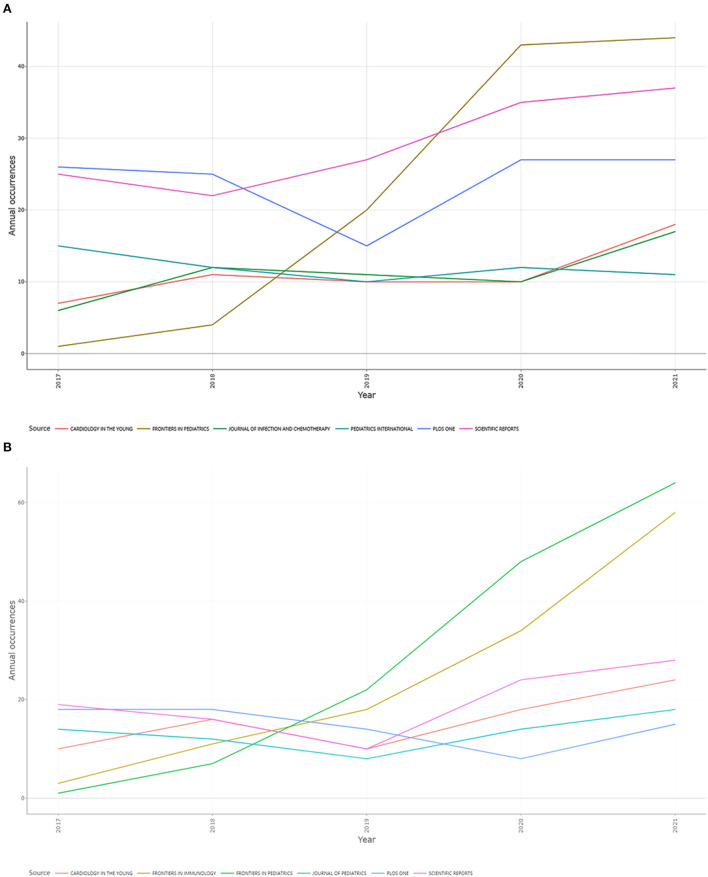
A line chart showing the annual trend of documents published in the top six journals of KD research (2017–2021): **(A)** WOS and **(B)** Scopus. The abscissa represents different years, the ordinate represents the number of documents published each year, and different color curves represent different journals.

When analyzing the total number of publications for the KD research, WOS had 2.50% of the papers published in *Scientific Reports*, followed by *PLoS ONE* (2.05%) and *Frontiers in Pediatrics* (1.91%), while Scopus had Frontiers in Pediatrics published the most papers (2.09%), followed by Frontiers in Immunology (1.82%) (as shown in Table 1). It is worth noting that the vast majority of the top 15 journals in terms of the number of published papers are not high impact factor journals, nor are they based on 2022 JCR^®^ journals in the Q2 category or higher. Figure 2 shows the publication trend for the top 6 journals in both databases over the last 5 years, with *Frontiers in Pediatrics* showing the most significant growth in both databases. For the WOS database, Scientific Reports is the second fastest growing journal, while the second fastest growing journal in Scopus is Frontiers in Immunology.

**Table 1:** The top 15 journals with the largest number of published papers about KD (2017–2021).

**Rank**	**WOS**							**Scopus**						
	**Sources**	**Country**	**NP (%),** ***n* = 5,848**	**TC**	**h-index**	**JCR^®^2022 impact factor**	**JCR^®^2022 category (quartile)**	**Sources**	**Country**	**NP (%),** ***n* = 6,804**	**TC**	**h-index**	**JCR^®^2022 impact factor**	**JCR^®^2022 category (quartile)**
1	Scientific reports	England	146 (2.50%)	1,855	22	4.996	Multidisciplinary sciences (Q2)	Frontiers in pediatrics	Switzerland	142 (2.09%)	1,193	16	3.569	Pediatrics (Q2)
2	PLoS ONE	USA	120 (2.05%)	1,187	18	3.752	Multidisciplinary sciences (Q2)	Frontiers in immunology	Switzerland	124 (1.82%)	2,797	26	8.786	Immunology (Q1)
3	Frontiers in pediatrics	Switzerland	112 (1.91%)	855	15	3.569	Pediatrics (Q2)	Scientific reports	England	97 (1.43%)	1,140	18	4.996	Multidisciplinary sciences (Q2)
4	Pediatrics international	Japan	60 (1.02%)	479	10	1.617	Pediatrics (Q4)	Cardiology in the young	USA	78 (1.15%)	310	9	1.023	Pediatrics (Q4)
5	Cardiology in the young	USA	56 (0.95%)	190	8	1.023	Pediatrics (Q4)	PLoS ONE	USA	73 (1.07%)	904	18	3.752	Multidisciplinary sciences (Q2)
6	Journal of infection and chemotherapy	Japan	56 (0.95%)	316	10	2.065	Infectious diseases (Q4); pharmacology and pharmacy (Q4)	Journal of pediatrics	USA	66 (0.97%)	1,276	19	6.314	Pediatrics (Q1)
7	Circulation journal	Japan	53 (0.91%)	649	13	3.35	Cardiac and cardiovascular systems (Q3)	International journal of molecular sciences	Switzerland	62 (0.91%)	1,055	17	6.91	Chemistry (Q1)
8	Medicine	USA	52 (0.89%)	190	8	1.817	Medicine, general and internal (Q3)	Pediatric rheumatology	England	58 (0.85%)	464	10	3.413	Pediatrics (Q2)
9	Journal of pediatrics	USA	50 (0.85%)	779	15	6.314	Pediatrics (Q1)	Pediatric infectious disease journal	UAS	50 (0.73%)	1,176	13	3.806	Pediatrics (Q1); infectious diseases (Q3); immunology (Q3)
10	Clinical and experimental nephrology	Japan	44 (0.75%)	354	10	2.617	Urology and nephrology (Q3)	Pediatrics international	Japan	50 (0.73%)	462	9	1.617	Pediatrics (Q4)
11	Pediatric rheumatology	England	44 (0.75%)	322	9	3.413	Pediatrics (Q2); rheumatology (Q3)	BMJ case reports	Japan	47 (0.69%)	180	6	N/A	N/A
12	Pediatric infectious disease journal	UAS	42 (0.71%)	423	11	3.806	Pediatrics (Q1); infectious diseases (Q3); infectious diseases (Q3);	BMC pediatrics	England	45 (0.66%)	317	11	2.567	Pediatrics (Q3)
13	Internal medicine	Japan	41 (0.70%)	142	6	1.282	Medicine, general and internal (Q4)	Medicine	USA	44 (0.65%)	865	14	1.817	Medicine, general and internal (Q3)
14	Frontiers in immunology	Switzerland	40 (0.68%)	694	13	8.786	Immunology (Q1)	International journal of rheumatic diseases	Australia	42 (0.62%)	488	9	2.558	Rheumatology (Q4)
15	International journal of molecular sciences	USA	39 (0.66%)	361	11	6.208	Biochemistry and molecular biology (Q1); chemistry, multidisciplinary (Q2)	Clinical rheumatology	England	41 (0.85%)	614	14	3.65	Rheumatology (Q3)

NP, number of papers; TC, total citation; JCR, journal citation report.

### Most influential authors and their collaborations networks

The top 10 most influential authors in the two databases varied widely. As shown in Table 2, Goodman SG, Kazunori Kataoka, Kimura Takeshi, and Marx Nikolaus had the highest influence in KD research in WOS using the *h*-index as a reference standard; while in Scopus, Wang Y, Burns JC and Newburger JW were the most influential. As for the number of published studies, (i) Shibagaki Yugo (NP = 70), (ii) Kuo Ho-Chang (NP = 61) and (iii) Burns JC (NP = 51) were the most contributing authors in the WOS database; however, in the Scopus database, (i) Wang Y (NP = 111), (ii) Zhang Y (NP = 95) and (iii) Li Y (*n* = 83) have the highest number of published studies.

**Table 2:** The h-index, total citations and numbers of published papers of the top 10 most influential and productive authors.

**WOS database**								**Scopus database**							
**Based on the h-index**				**Based on the number of published papers**				**Based on the h-index**				**Based on the number of published papers**			
**Authors**	**h-index**	**TC**	**NP**	**Authors**	**h-index**	**TC**	**NP**	**Authors**	**h-index**	**TC**	**NP**	**Authors**	**h-index**	**TC**	**NP**
Goodman SG	21	4,102	24	Shibagaki Yugo	14	746	70	Wang Y	20	1,617	111	Wang Y	20	1,617	111
Kataoka Kazunori	21	1,561	41	Kuo Ho-Chang	13	608	61	Burns JC	19	3,755	57	Zhang Y	18	1,383	95
Kimura Takeshi	21	2,744	33	Burns JC	18	3,194	51	Newburger JW	18	4,208	40	Li Y	14	815	83
Marx Nikolaus	21	4,672	23	Kashihara Naoki	15	1,193	46	Zhang Y	18	1,383	95	Kuo Ho-Chang	16	843	77
Lopes RD	20	5,105	21	Kataoka Kazunori	21	1,561	41	Arditi M	17	714	25	Zhang L	16	1,001	74
Kiss RG	19	3,938	21	Tremoulet AH	15	999	39	Tremoulet AH	17	1,214	45	Wang X	15	1,068	67
Burns JC	18	3,194	51	Yasuda Satoshi	10	263	38	Kuo Ho-Chang	16	843	77	Li X	14	636	66
Hagstrom Emil	18	3,844	20	Yamagata Kunihiro	15	721	36	Zhang L	16	1,001	74	Zhang J	15	785	65
Liberopoulos Evangelos	18	3,844	20	Kozuma Ken	13	2,095	36	Cimaz R	15	682	23	Liu Y	14	690	63
Newburger JW	17	3,629	34	Newburger JW	17	3,629	34	Wang X	15	1,068	67	Burns JC	19	3,755	57

TC, total citation; NP, number of published papers.

**Figure 3:**
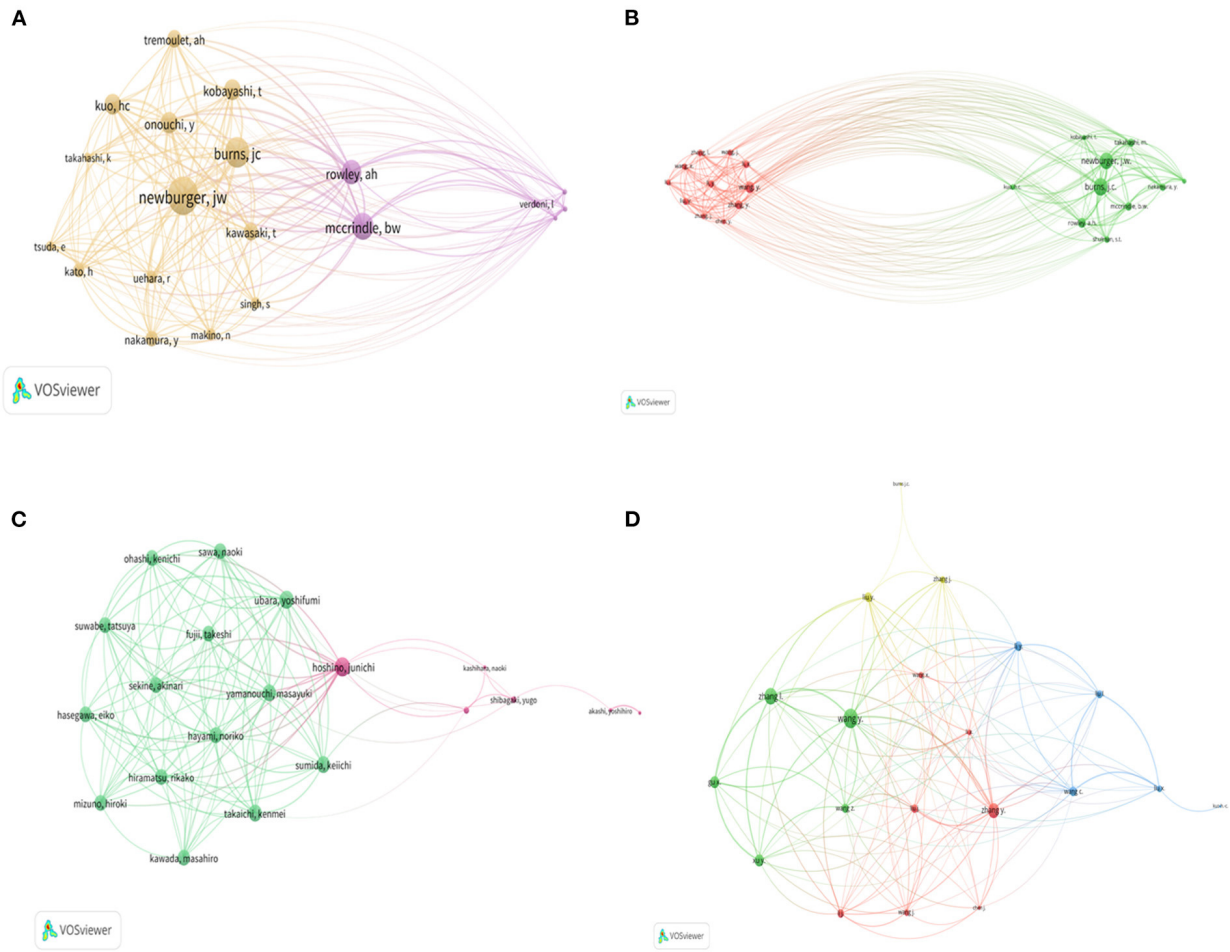
Analysis of the WOS and Scopus databases for the co-citation author network **(A, B)** and author collaboration network **(C, D)** involved in KD research from 2017 to 2021. Different colors represent different clusters, and the size of the network area is proportional to the link length.

Looking at the network of co-cited authors, the most co-cited authors in WOS were Newburger JW [link (L) = 19, link strength (LS) = 7,617, citation(C) = 1,241] and Mccrindle (*L* = 19, LS = 5,409, *C* = 1,114), while in Scopus, it was Burns JC (*L* = 20, LS = 40,839, *C* = 3,031) and Newburger JW (*L* = 20, LS = 34,633, *C* = 2,845) (Figures 3A, B). Among the retrieved WOS documents, the most important collaborations among researchers were Hoshino Junichi (*L* = 17, LS = 254, *C* = 185) and Ubara Yoshifumi (*L* = 16, LS = 255, *C* = 186). For the Scopus files, Wang L (*L* = 17, LS = 107, *C* = 1,538), Zhang L (*L* = 15, LS = 92, *C* = 935), and Zhang Y (*L* = 17, LS = 83, *C* = 1,234) were the most collaborative authors (Figures 3C, D).

### Most productive institutions and their cooperation

**Figure 4:**
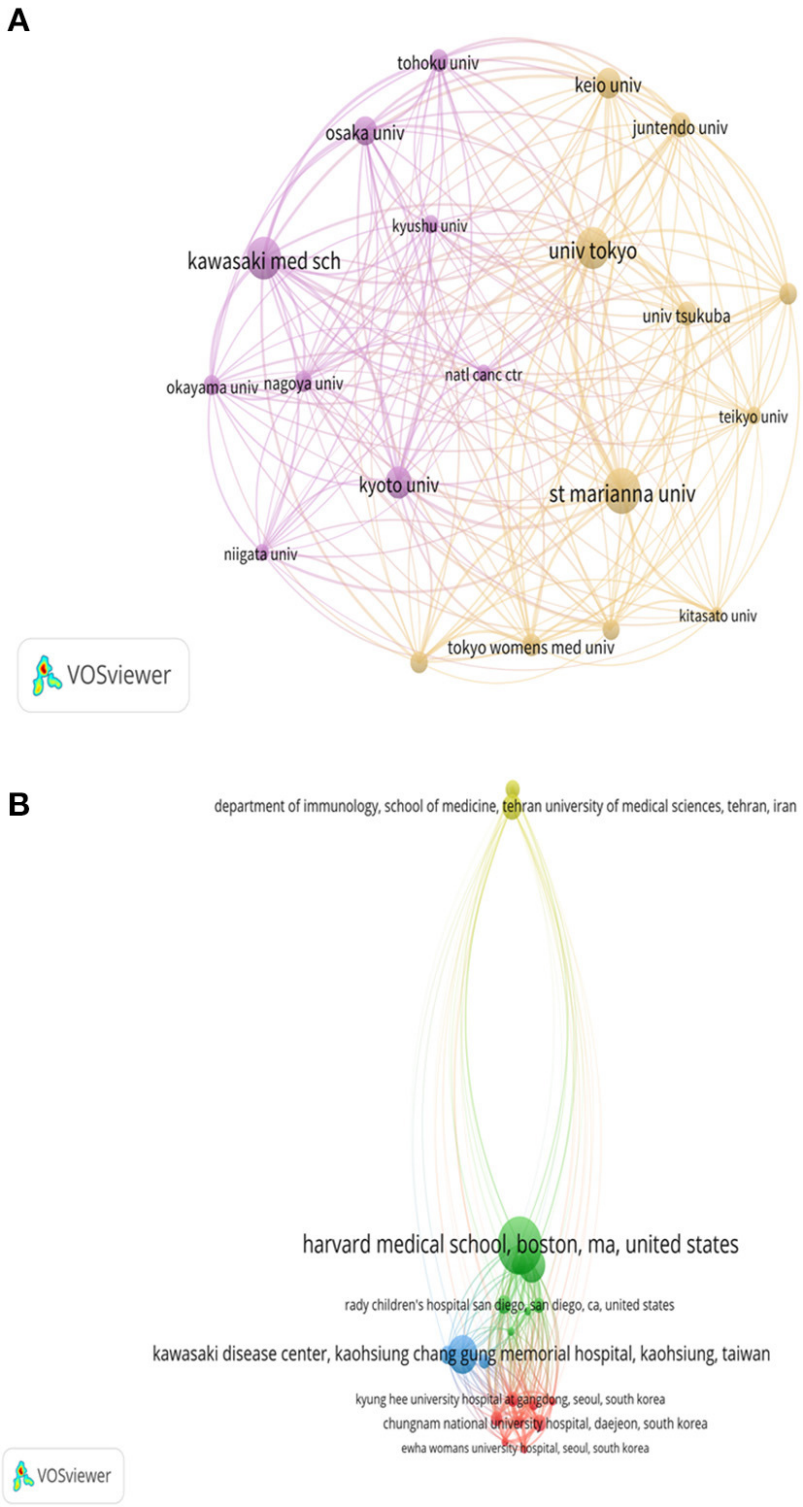
Collaboration networks between the most prominent research institutions in KD research: **(A)** WOS and **(B)** Scopus. Different colors belong to different clusters and the area of the dots is proportional to the inter-institutional collaboration.

The most productive institutions for KD research are shown in Table 3. For WOS files, three Japanese institutions, St. Marianna University, Kawasaki medical school, and The University of Tokyo, were the most productive institutions; while for Scopus, Harvard Medical School, University of California, and Tehran University of Medical Sciences were in the top three. Analysis of institutional collaboration revealed that St. Marianna University (*L* = 19, LS = 713, *C* = 6,591), Kawasaki Medical School (*L* = 19, LS = 657, *C* = 5,746), and The University of Tokyo (*L* = 19, LS = 643, *C* = 6,102) were the most collaborative institutions in the WOS (Figure 4A). While in Scopus, Harvard Medical School (*L* = 19, LS = 3,189, *C* = 3,266), Kaohsiung Chang Gung Memorial Hospital (*L* = 19, LS = 2,801, *C* = 327) and Boston Children's Hospital (*L* = 19, LS = 2,644, *C* = 272) were the most collaborative institutions (Figure 4B).

**Table 3:** Ten most productive affiliation in KD research in WOS and Scopus.

**Rank**	**WOS database**		**Scopus database**	
	**Affiliation**	**Articles**	**Affiliation**	**Articles**
1	St Marianna University School of Medicine	1,048	Harvard Medical School	177
2	Kawasaki Medical School	786	University of California	134
3	The University of Tokyo	753	Tehran University of Medical Sciences	128
4	Keio University	551	University of Toronto	120
5	Osaka University	531	China Medical University	105
6	Juntendo University	393	Capital Medical University	93
7	Kyoto University	384	Huazhong University of Science and Technology	91
8	Nippon Medical School	382	Chang Gung University College of Medicine	85
9	University of Tsukuba	329	Guangzhou Medical University	80
10	Yokohama-City University	328	Baylor College of Medicine	77

### World research production and collaboration

Table 4 shows the top ten major countries publishing on the topic of KD and their cooperative publishing proportion. In both databases, we can find that Japan, the United States and China are the countries with the highest number of published studies. For WOS, Japan leads in the number of both SCP and MCP. Interestingly, Canada has the highest rate of MCP (MCP Ratio = 0.562), followed by the UK (MCP Ratio = 0.556) and France (MCP Ratio = 0.507). While in Scopus, China was in first place in both SCP and MCP, and the highest percentage of highest rate of MCP was in the UK (MCP Ratio = 0.456).

**Table 4:** The top 10 major publishing countries with KD research and their cooperative publishing proportion.

**Rank**	**WOS database**				**Scopus database**			
	**Country**	**NP (%)**, ***n*** = **5,848**	**SCP**	**MCP**	**Country**	**NP (%)**, ***n*** = **6,804**	**SCP**	**MCP**
1	Japan	3,356 (57.4%)	2,961	395	China	1,350 (19.84%)	1,212	138
2	USA	631 (10.8%)	390	241	Japan	1,237 (18.18%)	1,094	134
3	China	563 (9.6%)	496	67	USA	1,101 (16.18%)	887	214
4	India	122 (2.1%)	109	13	Italy	332 (4.88%)	273	59
5	Italy	120 (2.1%)	92	28	India	226 (3.31%)	202	24
6	United Kingdom	108 (1.8%)	48	60	Korea	215 (3.16%)	193	22
7	Korea	101 (1.7%)	89	12	Turkey	200 (2.94%)	192	8
8	Canada	89 (1.5%)	39	50	United Kingdom	180 (2.65%)	98	82
9	Turkey	74 (1.3%)	69	5	Iran	175 (2.57%)	140	35
10	France	71 (1.2%)	35	36	Germany	161 (2.37%)	116	45

NP, total numbers of published paper; SCP, single country publication; MCP, multiple countries publication.

**Figure 5:**
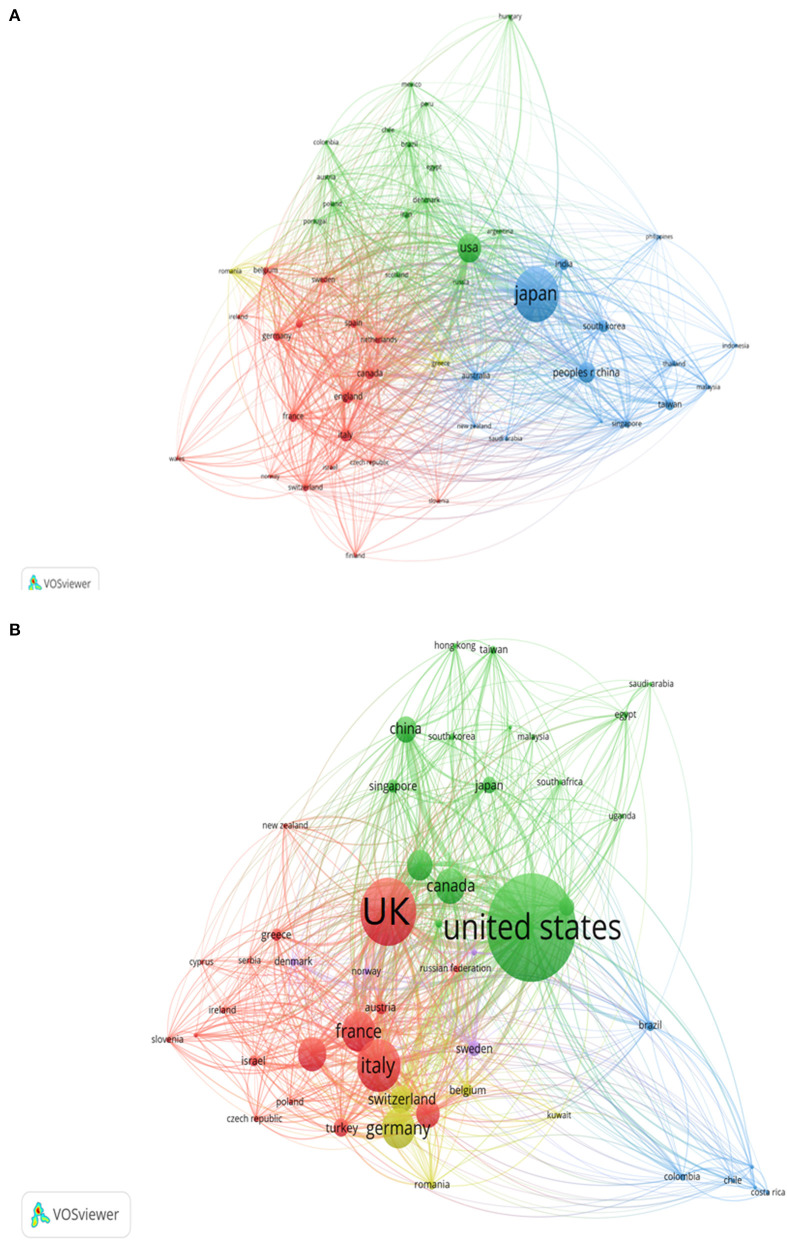
Network visualization of collaborations among countries of KD research in WOS **(A)** and Scopus **(B)**.

Figure 5 shows the network of collaborations among top 50 countries of KD research. And the top 50 countries could be roughly divided into several clusters using the “association strength” clustering method. For the WOS literature, these countries were broadly grouped into 4 clusters, with Japan (LS = 1,726, blue cluster), the USA (LS = 1,611, green cluster), and the UK (LS = 815, red cluster) being the most collaborative, while in Scopus, these countries were grouped into 5 clusters, suggesting that USA (LS = 1,157, green cluster), UK (LS = 723, red cluster), and Germany (LS = 430, yellow cluster) were the most critical in terms of KD research collaboration.

### Most cited literature

When analyzing the literature from WOS, the average citations per document and per document per year were 2.62 and 13.82, respectively; while the average citations per document and per document per year for the Scopus literature were 2.52 and 17.87, respectively. Among them, McCrindle et al. (5) entitled “Diagnosis, Treatment, and Long-Term Management of Kawasaki Disease: a Scientific Statement for Health Professionals From the American Heart Association,” with 1,400 total citations, was ranked first in WOS and was published in Circulation. And it was affiliated with American Heart Association Rheumatic Fever, Endocarditis, and Kawasaki Disease Committee of the Council on Cardiovascular Disease in the Young. While in Scopus, the top total citation is the review article entitled” Heart Disease and Stroke Statistics-2017 Update: A Report From the American Heart Association” by Benjamin et al. (33) published in Circulation. The article is part of the American Heart Association Statistics Committee and Stroke Statistics Subcommittee and has been cited a total of 6,057 times. The top 5 most cited documents on the topic of KD in the WOS and Scopus databases are shown in Table 5, and all of these studies have been cited more than 1,000 times.

**Table 5:** The top 5 most cited documents on KD (2017–2021).

**Rank**	**References**	**Title name**	**Document type**	**Journal name**	**DOI**	**Total citations**
**WOS database**						
1	Mccrindle et al. (5)	Diagnosis, treatment, and long-term management of kawasaki disease: a scientific statement for health professionals from the american heart association	Review article	Circulation	10.1161/CIR.0000000000000484	1,400
2	Garg et al. (34)	Hospitalization rates and characteristics of patients hospitalized with laboratory-confirmed coronavirus disease 2019—COVID-net, 14 states, march 1-30, 2020	Research article	Mmwr-morbidity and mortality weekly report	10.15585/mmwr.mm6915e3	1328
3	Verdoni et al. (6)	An outbreak of severe Kawasaki-like disease at the Italian epicenter of the SARS-CoV-2 epidemic: an observational cohort study	Research article	The Lancet	10.1016/S0140-6736(20)31103-X	1244
4	Feldstein et al. (35)	Multisystem inflammatory syndrome in U.S. children and adolescents	Research article	The new England journal of medicine	10.1056/NEJMoa2021680	1158
5	Gupta et al. (36)	Extrapulmonary manifestations of COVID-19	Review article	Nature medicine	10.1038/s41591-020-0968-3	1127
**Scopus database**						
1	Benjamin et al. (33)	Heart disease and stroke statistics-2017 update: a report from the American heart association	Review article	Circulation	10.1161/CIR.0000000000000485	6057
2	Benjamin et al. (37)	Heart disease and stroke statistics-2018 update: a report from the American heart association	Review article	Circulation	10.1161/CIR.0000000000000558	4240
3	Virani et al. (38)	Heart disease and stroke statistics-2020 update: a report from the American heart association	Review article	Circulation	10.1161/CIR.0000000000000757	3547
4	Mccrindle et al. (5)	Diagnosis, treatment, and long-term management of Kawasaki disease: a scientific statement for health professionals from the American heart association	Review article	Circulation	10.1161/CIR.0000000000000484	1647
5	Verdoni et al. (6)	An outbreak of severe Kawasaki-like disease at the Italian epicenter of the SARS-CoV-2 epidemic: an observational cohort study	Research article	The Lancet	10.1016/S0140-6736(20)31103-X	1360

### Literature survey of top 5 cited documents to KD research in WOS and Scopus database

Since the American Heart Association (AHA) published the first edition of its guidelines for the diagnosis, treatment, and long-term management of KD in 2004, more refined versions have been updated (5). McCrindle et al. (5) on behalf of the AHA, based on a review of the 2004 AHA scientific statement as well as the most recent version of the guidelines, with a focus on evaluating the most recent published studies at that time, the guidelines were revisions as necessary. The guidelines provide a new discussion of the epidemiology, genetics, pathogenesis, pathology, natural history, and long-term prognosis of KD, providing clinicians with new recommendations for the diagnosis, acute phase treatment, and long-term management of KD. However, the contributing expert group noted that there is still a lack of preventive measures for KD, while a small percentage of children with standardized treatment still develop coronary artery lesions (5).

In the same year, Benjamin et al. (33) published the 2017 edition of the AHA Heart Disease and Stroke Statistics Report. Since 2006, the AHA has included heart disease, stroke, and seven life factors associated for maintaining a healthy heart in its statistics and updated the report annually. Each revision of the Report will incorporate the most current and representative US health data, add updated scientific insights, and new sections. In section 16 of this updated Report, the findings of recent studies on KD were summarized, including the diagnosis of the disease, etiology and genetic mechanisms, and epidemiological data based on country, geography, and ethnicity. In addition, it identified late disease diagnosis, age at onset < 6 months, male gender, and Asian population as risk factors for the development of coronary artery lesions (33). The following year, the authors conducted a revision of the 2018 version. Based on the content of the old version, a new statement was added to section 14 of the report: as an acute inflammatory disease of acquired origin, KD is most common in East Asian populations (including Japan, Taiwan, and Korea), and its incidence is increasing year by year (37). Since then, Virani et al. also completed an update of the data for that year in 2020. This version added data on KD cases from the US mainland. USA had 6,000 KD discharges in 2016 (4,000 men and 2,000 women); and KD contributed to 5 patients' underlying mortality and 10 patients' all-cause mortality, respectively, in the 2017 US mortality data (38). Notably, the Statistical Update has been widely cited due to its scientific validity and completeness, with a total of more than 20,000 citations, and in the first 7 months of 2017 alone, the 2017 Statistical Update has been accessed more than 106,500 times (33, 37).

Since the outbreak of the novel coronavirus SARS-CoV-2, several national health agencies have been involved in monitoring and analyzing the outbreak data. The Garg et al. (34) application COVID-19-Associated Hospitalization Surveillance Network (COVID-NET), led by the CDC COVID-NET team, conducted population-based disease data surveillance. The report included data on disease in patients hospitalized for COVID-19 in March 2020, and the analysis noted the highest prevalence in older adults aged ≥65 years, while the vast majority of hospitalized patients (~90%) had underlying disease, with chronic conditions such as obesity, hypertension, diabetes, and cardiovascular disease being prevalent (34).

Gupta et al. (36) conducted a comprehensive review of the pathophysiological and clinical effects of COVID-19 on various organ systems using the extra-pulmonary manifestations of SARS-CoV-2 as the object of analysis. This review summarizes the viral pathophysiology and virulence mechanisms of SARS-CoV-2, and pioneers the possible clinical manifestations and disease mechanisms in multiple extra-pulmonary organ systems (including hematological, cardiovascular, renal, gastrointestinal, hepatobiliary, endocrine, ocular and neurological, and skin) and special disease conditions in humans, including children and pregnant women, separately. In the section on pediatric-associated SARS-CoV-2 infection, this review summarized the definition of MIS-C and potentially useful therapeutic measures and suggest that the underlying mechanism in most children with MIS-C may arise from an acquired immune response in the body rather than direct SARS-CoV-2 injury (36).

To assess the incidence and clinical characteristics of patients with KD-like illness diagnosed during the SARS-CoV-2 epidemic, a cohort study was conducted by Verdoni et al. (6) in Bergamo Province, Italy. The study reviewed information on the disease in children who met the diagnostic criteria for KD from January 1, 2015 to April 20, 2020, and grouped these children according to the timing of the local SARS-CoV-2 outbreak. Ultimately, it was found that the incidence of KD in the area increased dramatically by 30-fold after the virus epidemic, and most of the children diagnosed showed positive results for SARS-CoV-2. These children who tested positive tended to be relatively older, had a higher incidence of cardiac complications, and had more severe disease manifestations (6). Although this is a small sample case series study, it has been widely cited because it explores the potential relationship between the surge of Kawasaki-like disease and the SARS-CoV-2 epidemic, which has far-reaching clinical implications. Meanwhile, Feldstein et al. (35) summarized the epidemiological and clinical characteristics of a total of 186 children with MIS in 26 states in the United States and indicated that SARS-CoV-2-associated MIS-C can be life-threatening in a population of healthy minors. This study found that ~40% of the 186 children presented with Kawasaki-like clinical features, and 80% of children with MIS were placed in intensive care. In terms of pharmacological treatment, nearly half of the children required vasoactive drugs and immunomodulators were commonly applied to fight multisystem inflammation.

### Keywords analysis and topic trends

**Figure 6:**
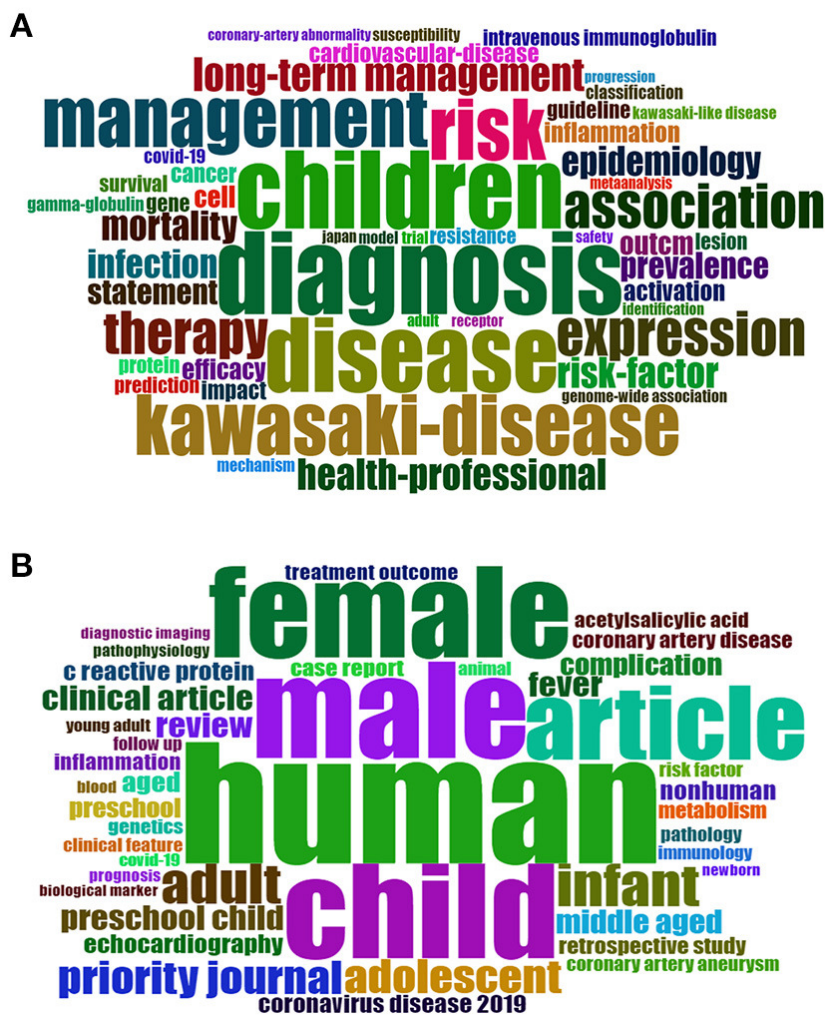
Visualized word-clouds of keywords plus in WOS **(A)** and Scopus **(B)** (Only show the first 50 keywords).

Text analysis revealed that the WOS documents had 10,458 keywords plus and 11,469 author keywords, while the Scopus documents had 25,423 keywords plus and 11,078 author keywords. The direction and themes of KD research can be identified to further understand the current trend of the discipline by analyzing the keyword distribution. It is worth noting that keywords plus are essential words for exploring a scientific field and they often appear in the title of the article reference rather than in the title of the article (39). Supplementary Table S1 shows the top 10 most frequently occurring keywords plus and author keywords. Since this topic is aimed at KD research, the author keywords appeared most frequently with “Kawasaki disease,” followed by “COVID-19,” “SARS-CoV-2” and “children.” In addition, we can find the top 10 author keywords mainly related to novel coronaviruses, disease mechanisms (vascular, inflammation, and mis-c) and their therapeutic measures (intravenous, immunoglobulin). As for keywords plus, it is mainly related to the diagnosis and management of the disease (Figure 6). The size and centering of the keyword in the Word Cloud reflects its frequency and magnitude.

**Figure 7:**
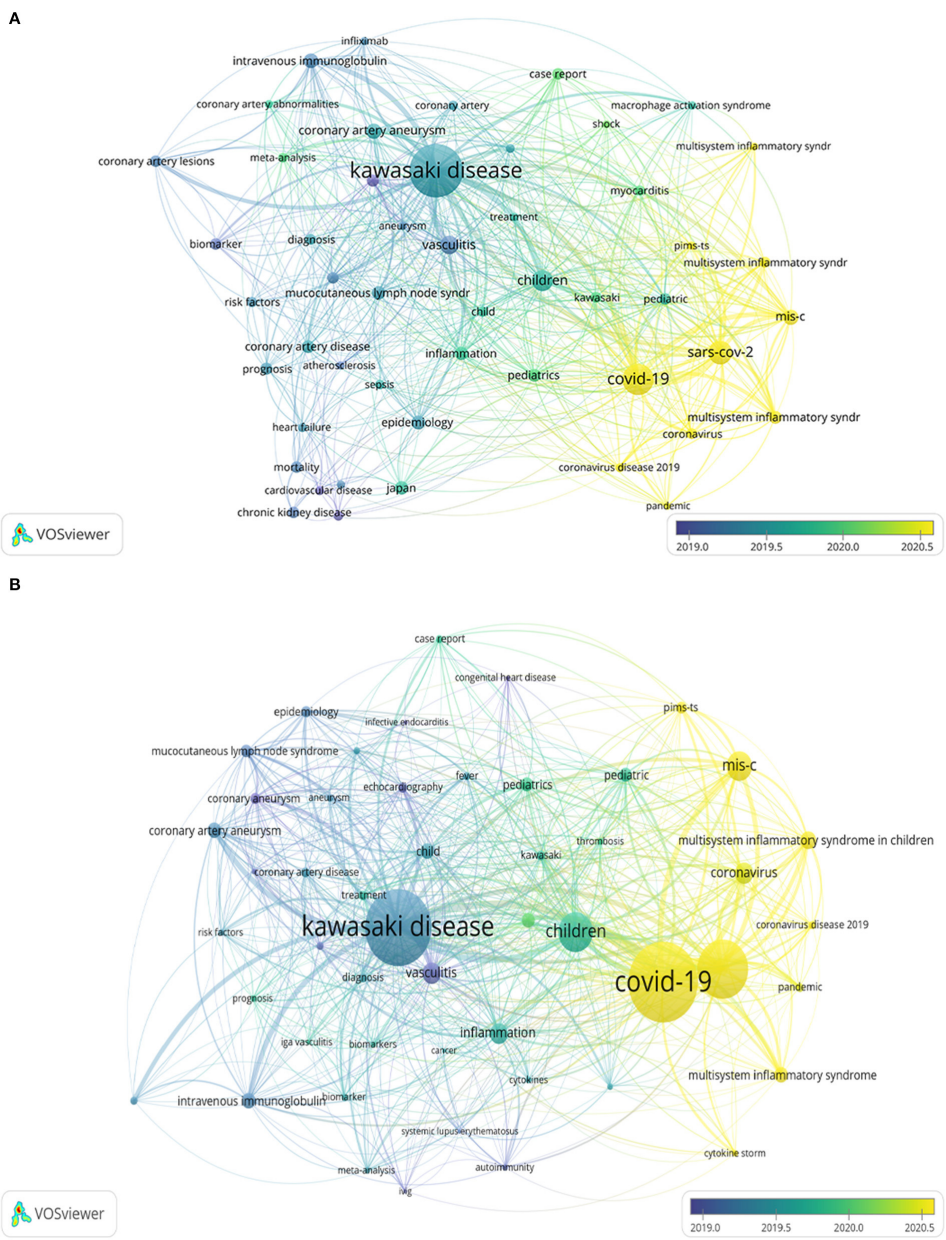
Time-based visualization of keywords variation: **(A)** WOS and **(B)** Scopus.

By applying VOSviewer, the frequency of occurrence of keywords and the process of change over time were visualized. The top 50 author keywords with a frequency of at least 5 occurrences were analyzed for both databases (Figure 7). When the minimum cluster size was set to 10, keywords in the WOS literature were classified into four categories: (1) Novel coronavirus-associated multisystem inflammatory syndrome in children; (2) cardiac, renal, and vascular-related prognosis of KD; (3) symptoms of KD; and (4) diagnosis and treatment of KD; while keywords in the Scopus literature were classified into three categories: (1) Novel coronavirus-associated multisystem inflammatory syndrome in children associated multisystem inflammatory syndrome; (2) diagnosis, mechanism, and treatment of KD; and (3) cardiac-related complications of KD (Supplementary Table S2). The purple author keywords in Figure 7 appear earliest, while the yellow author keywords appear latest. The keywords “COVID-19,” “multisystem inflammatory syndrome,” and “pandemic” are the most recent in both data. Therefore, we can use the latest emerging keywords to understand the frontier hot spots of KD research.

### Sankey diagrams: Three field plots on KD research

**Figure 8:**
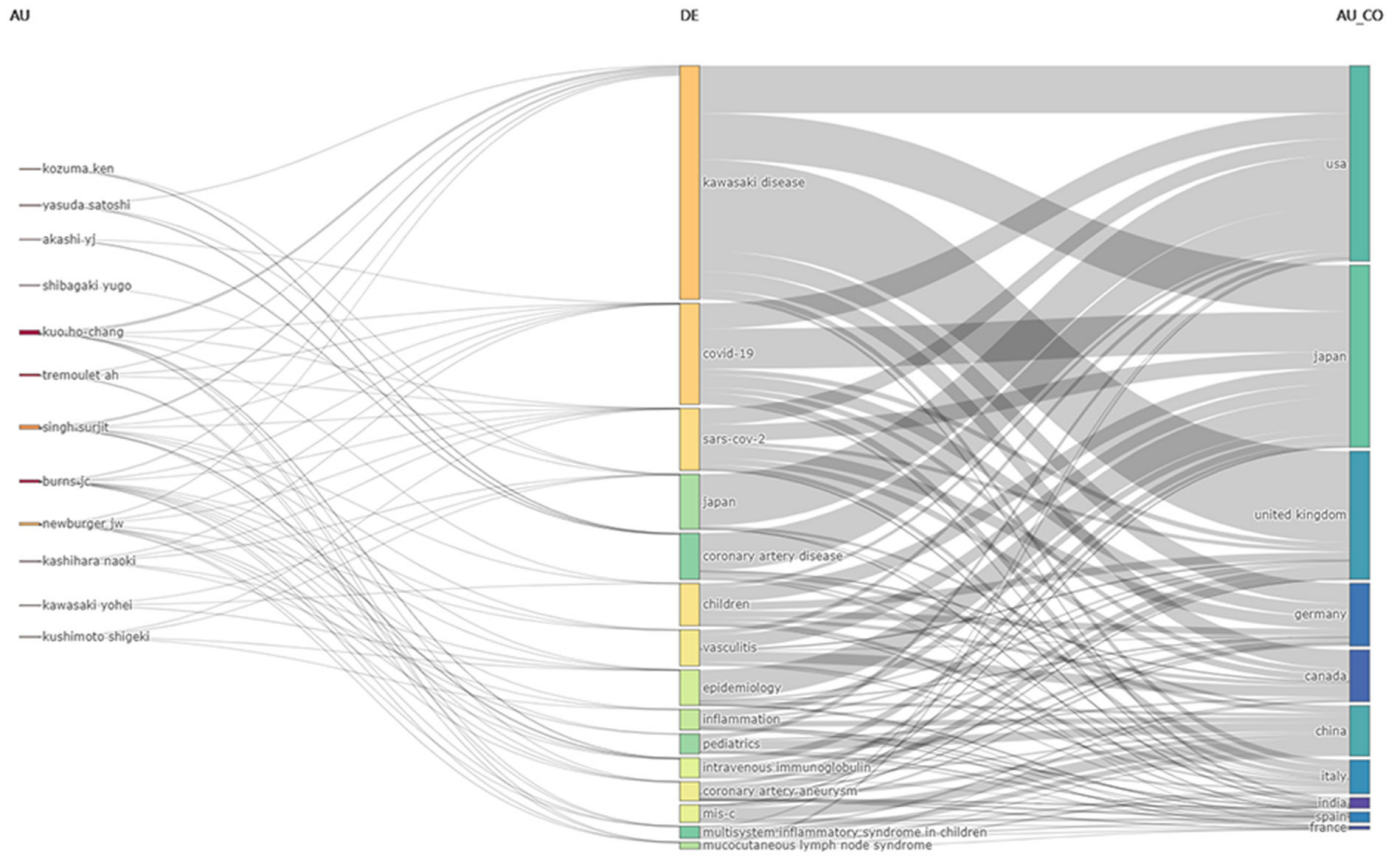
Three field plots on KD research. The picture represents the authors who have made the greatest contribution to KD research in the past 5 years (2017–2021), the author keywords and the inflow and outflow of countries. AU, author; DE, author Keywords; AU_CO, countries.

The Biblioshiny three-field plot represents the communication relationships between elements by combining rectangles representing different elements and connecting lines between rectangles, and is often used to visualize the connections between literature sources, countries/regions, affiliations, keywords, primary authors, cited sources, and author keywords. Where the larger the rectangle, the more communication between multiple components (40). Figure 8 shows a schematic representation of studies in the KD research literature on the relationship between authors (left), keywords (center), and authors' countries (right). The analysis identified which keywords of the KD study were most frequently used by different authors and countries. The analysis of the most frequently used keywords, source authors and countries suggested that five authors Kuo HC, Tremoulet AH, Singh Surjit, Burns JC, Newburger JW, five keywords, namely “Kawasaki disease,” “covid-19,” “SARS-CoV-2,” “Japan,” and “coronary artery disease,” which originated from four countries, USA, Japan, UK, and Germany.

## Discussion

Using bibliometric analysis methods and visualization software as technical support, this study explored the research trends and hotspots in the field of KD from 2017 to 2021. The number of papers published in this research area has continued to increase in the last 5 years, and the type is dominated by Article. Taking 2019, the year when the novel coronavirus outbreak epidemic started, as the boundary, the number of KD papers published grew even faster, and the growth characteristics of papers in different databases were not fully consistent, which shows that this research field may still have more scientific output in the coming years. The citation volume curve showed that the average annual citation volume was essentially flat during 2017–2019, while a significant fluctuation was observed in 2020. The apparent increase in citation volume is consistent with the findings of another Google trend study on COVID-19 and Kawasaki disease (41). This may be related to the continued discovery of symptoms similar to Kawasaki disease following the COVID-19 outbreak (41, 42).

This study found that both WOS and Scopus database publications on KD research originated mainly three countries, Japan, USA, and China, with Japan and USA having the highest total number of citations in the WOS and Scopus databases, respectively. This result is not surprising, as Japan was the first country to identify and intensively study KD (43). KD has a high prevalence throughout Northeast Asia (38). In China, the overall prevalence of KD appears to be on the rise, with data from questionnaire surveys in Beijing and Shanghai in 2017 showing a prevalence of 46.3–55.1 per 100,000 Chinese children under 5 years of age, and 28.58–60.08 per 100,000 children younger than 5 years of age in Taiwan (44, 45). This has gradually drawn attention to KD in this populous country (46). In China, the first expert consensus was written by the Shaanxi Provincial Diagnosis and Treatment Center of Kawasaki Disease in 2021 to regulate the use of IVIG in KD (47). The above reasons have led to more in-depth research in these countries.

Of the top 10 most productive countries, 70% were developed countries, which may be related to the fact that these countries have access to more adequate research fund support. When analyzing national collaboration networks, KD research is most collaborative in developed countries such as Japan, USA, and the United Kingdom, reflecting the central position of these countries in the field of KD. However, none of the African countries have been included in these top productivity rankings. A study showed a map of the global incidence of KD, with African regions represented as gray areas due to lack of available data (48). However, a study from the African country of Egypt showed that missed undiagnosed or untreated KD may be prevalent in that country (49). The lack of awareness of KD in African countries and the poor surveillance mechanisms in government health institutions may have contributed to this result. There is a need for us to provide the necessary financial support and assistance to African countries in the surveillance and treatment of KD.

In the WOS database, the most cited article was written by McCrindle et al. (5); this was written to revise previous American Heart Association guidelines. This article provides more scientific and novel guidelines for the diagnosis and treatment of KD and highlights that individualized treatment plans should be developed for different patients (5). In the Scopus database, “Heart Disease and Stroke Statistics-2017 Update: A Report From the American Heart Association” by Benjamin et al. (33) is the most cited. Analysis of the most cited literature from both databases shows that KD has been of great interest in the field of pediatrics, especially in terms of diagnosis, etiologic mechanisms and treatment of the disease. Since the COVID-19 epidemic, SARS-CoV-2 infection has been implicated as an infectious factor contributing to the dramatic increase in KD. Exploring the pathological mechanisms, clinical features and therapeutic measures of Kawasaki-like diseases associated with SARS-CoV-2 infection has become a hot research topic in recent years.

When evaluating the most productive journals based on number of publications, ie was found that not all of the top 15 journals in both databases were Q2+ journals base on 2022JCR ^®^ and not all had high impact factors. For the WOS database, Scientific Reports ranked first in terms of number of publications, total citations and h-index, while in the Scopus database, Frontiers in Pediatrics was the highest contributor. Frontiers in Pediatrics has the fastest growth rate in the number of published papers in the last 5 years. However, analysis of the main source journals of cited literature reveals that most of the cited literature on KD research originates from high impact factor journals, such as *Circulation, The New England Journal of Medicine*, and *Lancet*. We speculate that it is more difficult to publish KD research papers in high impact factor journals, but they can receive more attention and citations than publishing them in low impact factor journals (50). And, in fact, an essential and important factor in order for an individual paper to obtain more citations is the effort put into writing the paper (51). Therefore, we believe that the quality of a research paper cannot be judged solely on the basis of the impact factor of the journal.

Based on the h-index and the number of papers published, this study analyzed the most influential and most productive authors, respectively. In addition, the most cited authors from the co-cited author network observed for both databases were Newburger JW and McCrindle BW and Burns JC, suggesting that their research results are widely followed and recognized. The author collaboration network shows that the author team with two authors, Hoshino Junichi and Ubara Yoshifumi, as the main leaders and the research teams led by Wang L, Zhang L and Zhang Y, respectively, are the most closely collaborated. The above information will help future researchers working in the field of KD research to more accurately and quickly identify influential research teams and to consult relevant individuals in a targeted manner.

In the WOS database, the top three most productive institutions were all from Japanese institutions and showed the highest level of collaboration and formed collaborative networks with other institutions. For the Scopus database, it is the US institutions that have the highest output, and the inter-institutional collaboration tips are dominated by US institutions. This implies that in the field of KD, two developed country research institutions, Japan and the United States, hold more research resources and are in the leading position in international research. And the analysis of global inter-country collaboration showed that Japan, the United States and the United Kingdom were the most cooperative countries.

Keyword analysis reveals the most common author keywords and the temporal sequence of keyword occurrences. “Kawasaki disease,” “COVID-19,” “SARS-CoV-2,” and “children” were the most common keywords. Time-based keyword change analysis showed that the keywords “COVID-19,” “multisystem inflammatory syndrome,” and “pandemic” have appeared only recently. KD mainly causes systemic vascular inflammation mainly in infants and children (30). Our results showed a high frequency of the keyword “children,” and the papers included in the analysis were mainly from the pediatric population. In the United States, the Centers for Disease Control proposed in May 2020 a new disease, MIS-C, which is associated with COVID-19 (52). These children share common features with KD, i.e., mild pulmonary signs but marked and severe systemic inflammation (53). The high frequency of “Kawasaki disease” and “COVID-19/SARS-CoV-2” also suggests an important association between Kawasaki disease and COVID-19. SARS-CoV-2 may be a factor in triggering Kawasaki disease (54). Such children with SARS-CoV-2-induced KD have an older age of onset and are characterized clinically by the development of myocarditis (26). Verdoni et al. (32) also revealed that Italian patients with COVID-19 can develop a more severe symptomatic Kawasaki-like disease, and these patients often require adjuvant glucocorticoid therapy. By analyzing the co-linear relationships of the keywords, we classified the authors' keywords. Notably, the keywords in both databases can be broadly classified into the categories of novel coronavirus-associated multisystem inflammatory syndrome in children, diagnosis and treatment of KD, and complications associated with KD, which may be a key direction for future KD research.

Looking at the results of the data analysis throughout the study, it is easy to see that there are indeed major differences between the WOS and Scopus databases. Previous studies also confirm our findings that Scopus provides 20% more coverage of citation information than Web of Science and that Scopus has a broader range of journals (55). When performing bibliometric analysis, searching only one of the databases may result in missing important data, and joint searching is recommended to broaden the scope of the search.

Although this study is the first bibliometric analysis of studies with KD as a topic in the last 5 years. And the research hotspots in the field were explored, which is informative. However, there are some limitations, such as the inclusion of only English articles in the analysis, which may have overlooked important research information in other languages. In this study, factors such as country, institution, journal, author, and keywords were analyzed at a macro level. However, the influence of relevant medical policies, support funds, and government agencies on KD research needs further investigation.

## Conclusion

This bibliometric analysis summarizes for the first time the research progress in KD (2017–2021), providing a qualitative and quantitative assessment of the bibliometric information on KD research. In this field, researchers mainly from Japan and USA are dominant, followed by China. It is recommended to pay close attention to the latest hotspots, such as “COVID-19” and “multisystem inflammatory syndrome.” These results provide a more intuitive and convenient way for researchers to obtain the latest information on KD research.

## Data availability statement

The original contributions presented in the study are included in the article/Supplementary material, further inquiries can be directed to the corresponding author.

## Author contributions

WT was responsible for most of the work including literature screening, data extraction, statistical analysis, paper writing, manuscript submission, and revision. LJ and YW were responsible for literature screening, image creation, and other necessary support. WL provided project proposal design, guidance, and financial support for this work. All authors contributed to the article and approved the submitted version.

## Supplementary material

Supplementary Table S1 The most frequent keywords and their frequency.

Supplementary Table S2 Author keyword cluster of KD research.
