# Supplementary material for: A global bibliometric analysis on Kawasaki disease research over the last 5 years (2017–2021)
Source: Front Public Health. 2023 Jan 10;10:1075659. doi: 10.3389/fpubh.2022.1075659 (PMC9871775; doi:10.3389/fpubh.2022.1075659)
Supplement: Supplementary Table S1 — The most frequent keywords and their frequency. [file Table_1.DOCX]

**Table S1.** The most frequent author keywords and their frequency.

| Rank | WOS Database | | Scopus Database | |
| --- | --- | --- | --- | --- |
|  | Author Keywords | Occurrences | Author Keywords | Occurrences |
| 1 | Kawasaki disease | 1086 | Kawasaki disease | 1171 |
| 2 | covid-19 | 378 | covid-19 | 791 |
| 3 | sars-cov-2 | 213 | sars-cov-2 | 420 |
| 4 | children | 177 | children | 412 |
| 5 | vasculitis | 130 | inflammation | 245 |
| 6 | coronary artery aneurysm | 93 | vasculitis | 235 |
| 7 | mis-c | 92 | child | 133 |
| 8 | intravenous immunoglobulin | 84 | intravenous immunoglobulin | 133 |
| 9 | epidemiology | 79 | mis-c | 131 |
| 10 | inflammation | 73 | coronary artery aneurysm | 120 |
