# Supplementary material for: A global bibliometric analysis on Kawasaki disease research over the last 5 years (2017–2021)
Source: Front Public Health. 2023 Jan 10;10:1075659. doi: 10.3389/fpubh.2022.1075659 (PMC9871775; doi:10.3389/fpubh.2022.1075659)
Supplement: Supplementary Table S2 — Author keyword cluster of KD research. [file Table_2.DOCX]

**Table S2.** Author keywords clusters in the field of Kawasaki disease.

| Cluster | WOS Database | Scopus Database |
| --- | --- | --- |
|  | Keywords | Keywords |
| 1 | children; coronavirus; covid-19; Kawasaki; macrophage activation syndrome; multisystem inflammatory syndrome in children; pandemic; myocarditis; pediatrics; epidemiology | coronavirus; coronavirus disease 2019; covid-19; cytokine storm; mis-c; multisystem inflammatory syndrome; multisystem inflammatory syndrome in children; myocarditis; pandemic; pims-ts; sars-cov-2; thrombosis; pediatrics; Kawasaki |
| 2 | atherosclerosis; cardiovascular disease; chronic kidney disease; coronary artery disease; diabetes; heart failure; hypertension; echocardiography; mortality; risk factors; sepsis; prognosis | Autoimmunity; biomarker; cancer; coronary artery lesions; cytokines; diagnosis; IgA vasculitis; Inflammation; Intravenous immunoglobulin; IvIg; macrophage activation syndrome; meta-analysis; prognosis; systemic lupus erythematosus; treatment; vasculitis |
| 3 | aneurysm; coronary artery; coronary artery aneurysm; fever; inflammation; mucocutaneous lymph node syndrome; shock; vasculitis; child | aneurysm; atherosclerosis; case report; child; children; congenital heart disease; coronary artery; coronary artery disease; echocardiography; epidemiology; fever; infective endocarditis; Kawasaki disease; mucocutaneous lymph node syndrome; myocardial infarction; pediatric; risk factors |
| 4 | biomarker; coronary artery abnormalities; coronary artery lesions; diagnosis; infliximab; intravenous immunoglobulin; treatment |  |
